# Novel Bilayer Micropyramid Structure Photonic Nanojet for Enhancing a Focused Optical Field

**DOI:** 10.3390/nano11082034

**Published:** 2021-08-10

**Authors:** Shaobo Ge, Weiguo Liu, Jin Zhang, Yuetian Huang, Yingxue Xi, Pengfei Yang, Xueping Sun, Shijie Li, Dabin Lin, Shun Zhou, Yechuan Zhu, Wenli Li, Yiting Yu

**Affiliations:** 1 Shaanxi Province Key Laboratory of Thin Films Technology and Optical Test, School of Optoelectronic Engineering, Xi’an Technological University, Xi’an 710032, China; geshaobo@126.com (S.G.); j.zhang@xatu.edu.cn (J.Z.); huangyuetian@xatu.edu.cn (Y.H.); xiyingxue@163.com (Y.X.); pfyang@xatu.edu.cn (P.Y.); xuepingsun@xatu.edu.cn (X.S.); lishijie@xatu.edu.cn (S.L.); dabinlin@xatu.edu.cn (D.L.); zsemail@126.com (S.Z.); zyc_xatu@126.com (Y.Z.); 2 Research & Development Institute of Northwestern Polytechnical University in Shenzhen, Shenzhen 518057, China; Wenlili_nwpu@163.com (W.L.); yyt@nwpu.edu.cn (Y.Y.); 3 College of Mechanical Engineering, Ningbo Institute of Northwestern Polytechnical University, Northwestern Polytechnical University, Xi’an 710072, China

**Keywords:** micro-optics, thin film optics, photonic nanojet, multilayer design, microfabrication

## Abstract

In this paper, synthetically using refraction, diffraction, and interference effects to achieve free manipulation of the focused optical field, we firstly present a photonic nanojet (PNJ) generated by a micropyramid, which is combined with multilayer thin films. The theory of total internal reflection (TIR) was creatively used to design the base angle of the micropyramid, and the size parameters and material properties of the microstructure were deduced via the expected optical field distribution. The as-designed bilayer micropyramid array was fabricated by using the single-point diamond turning (SPDT) technique, nanoimprint lithography (NIL), and proportional inductively coupled plasma (ICP) etching. After the investigation, the results of optical field measurement were highly consistent with those of the numerical simulation, and they were both within the theoretical calculation range. The bilayer micropyramid array PNJ enhanced the interference effect of incident and scattered fields; thus, the intensity of the focused light field reached 33.8-times that of the initial light, and the range of the focused light field was extended to 10.08λ. Moreover, the full width at half maximum (FWHM) of the focal spot achieved was 0.6λ, which was close to the diffraction limit.

## 1. Introduction

The manipulation of the optical field without restraint has always been an ultimate pursuit in optics. The microstructure/nanostructure with the advantages of tailoring the light properties has become one of the impressive approaches to achieve the above goal [1,2,3]. The photonic nanojet (PNJ) is a typical example of realizing a focused optical field through the microstructure [4,5].

The most outstanding characteristics of the PNJ are the subwavelength focusing [6,7], the long focus range [8,9], and being easily integrated with other technologies [10,11], which can bring a wide range of opportunities for the practical application of the PNJ in nanolithography [12,13], nanoscopy [14,15], all-dielectric switching [16,17], optical trapping and manipulation [18,19,20], etc.

According to literature reports, the spatial shape of the microstructure plays an important role in the features of the PNJ [21,22]. Thus, microstructures with various spatial forms [23] (sphere [24], cylinder [25], ellipse [26], cone [27], pyramid [28], cuboid [29], etc.) have been actively investigated.

Recently, various types of functional devices with micropyramids have been proposed [28,30]. In 2014, the formation of the PNJ by micropyramids was reported for the first time [31]. Later on, the relationship between the geometry size and optical field distribution of the micropyramid array was theoretically analyzed by the finite-difference time-domain (FDTD) method [32,33,34,35]. We note that the focusing properties of the pyramidal particles depend on the its orientation vs. the illuminating wave [31,36]. In 2018, the first direct experimental observation of the 2D-PNJ generated from a saw-tooth phase diffraction grating was reported [37]. All these research works were based on micropyramids with a homogeneous material. Obviously, the PNJ properties of the micropyramids are worth further investigation when the multilayer structure and gradient contour are considered.

Unlike the previous studies, we introduced a multilayer structure into the micropyramid. Considering the morphological characteristics of the pyramid structure, it is convenient to introduce the total internal reflection (TIR) theory to the design of the multilayer microstructure. The best-known application of total internal reflections is in optical fibers, which are used in communication cables to carry signals over long distances with little attenuation [38]. Another application demonstrated a TIR geometry in a near-field imaging system, in which a spatial modulator was formed by combining a light-sensitive silicon wafer with a prism. This unique combination allowed images of the sample structure to be reconstructed with a subwavelength lateral resolution [39]. The application of TIR combined with Light-Emitting Diodes (LEDs) effectively enhances the efficiency of light energy, which utilizes the evanescent wave in a TIR setup coupled with a conductive interface [40]. Besides, the design of the following applications involves TIR theory, for example the polarizing prism [41], total internal reflection fluorescence microscopy [42], the total reflection prism laser gyro [43], etc. These works inspired us to introduce TIR theory in the design of the bilayer micropyramid structure photonic nanojet.

At present, the design and manufacturing methods of the micropyramid combined with multilayer thin films have not been reported yet. Here, the bilayer micropyramid PNJ (BMP-PNJ) is proposed. The theory of total internal reflection (TIR) and the method of derivation via the expected focused spot size were creatively used to design the structure of the micropyramid. The phenomenon of the BMP-PNJ was investigated by using high-resolution FDTD numerical modeling. Furthermore, the as-designed bilayer micropyramid array was fabricated, and based on the analysis of the optical field distribution characteristics, the physical principles of the BMP-PNJ were investigated and discussed in detail.

## 2. Materials and Methods

### 2.1. Theory and Design

**Figure 1:**
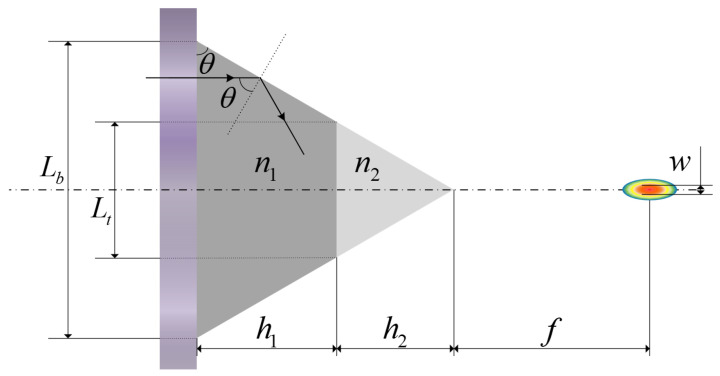
The main parameters of the bilayer micropyramid photonic nanojet.

The BMP-PNJ follows the Mie scattering theory, which defines the range of the refractive index values [21,28,30]. For the first time, the theory of total internal reflection [44] was introduced to design the microstructure of a photonic nanojet. We also back-deduced the size relationship of the bilayer micropyramid according to the required light spot characteristics, which is different from the previous direct calculation of the light field distribution of the PNJs. The main parameters of the BMP-PNJ are shown in Figure 1. $L_{b}$ and $L_{t}$ are the base side length of the bottom and top structure. $h_{1}$ and $h_{2}$ are the height of the bottom and top structure. $n_{1}$ and $n_{2}$ are the refractive index of the bottom and top structure. *f* is the focal length, which refers to the length from the apex of the bilayer micropyramid to the point of the maximum intensity ($I_{max}$). *w* is the full width at half maximum (FWHM).

Based on the theorem of total internal reflection, light emitted from the inside of the microstructure to the air satisfies Equation (1).

(1) $$n_{1}\cdot \sin\;\theta_{c}=n_{air}\cdot \sin\left(90^{\circ }\right)$$

Then, we have:

(2) $$\theta_{c}=\arcsin\left(\frac{1}{n_{1}}\right)$$

$n_{1}$ and $n_{air}$ are the refractive index of the micropyramid and air, respectively. $\theta_{c}$ is the critical angle. θ is the incident angle. It can be clearly seen that the base angle of the micropyramid is equal to the incident angle θ. The aspect ratio of the microstructure can be calculated by the following formula.

(3) $$\tan\theta =\frac{\left(h_{1}+h_{2}\right)}{L_{b}/2}$$

If θ > $\theta_{c}$, the incident light suffers total internal reflection; none of it is transmitted. The value of $n_{1}$ ranges from 1.46 (silicon oxide film) to 2.04 (silicon nitride film), which is determined by the plasma enhanced chemical vapor deposition (PECVD) method [45]. When materials with different refractive indexes are selected, different critical angles of total reflection can be obtained. Figure 1 shows the main parameters of the bilayer micropyramid photonic nanojet.

The aspect ratio of the microstructure can be calculated by:

(4) $$r=\frac{h_{1}+h_{2}}{L_{b}}$$

If silicon oxide film is selected, then $\theta >\theta_{c}=43.2^{\circ }$, and we have r≥0.47. If silicon nitride film is selected, then $\theta >\theta_{c}=29.4^{\circ }$, and we have r≥0.28.

Resulting from a limitation of our manufacturing capacity, the microstructure aspect ratio of the SiO_2_ and SiN_*x*_ materials was about 0.43. Therefore, we chose the silicon nitride film as the bottom layer to ensure the total internal reflection effect.

The BMP-PNJ can achieve subwavelength focusing, which brings the possibility to deduce the relationship among the size parameters in reverse. The FWHM *w* is determined by the first-order approximation of a focusing point spread function, which means *w* can be expressed by the equations below:

(5) $$w=\frac{\lambda }{2\cdot NA}$$

(6) $$NA=\sin\left[\tan^{-1}\left(\frac{L_{b}/2}{h_{1}+h_{2}+f}\right)\right]$$

The bilayer micropyramid photonic nanojet maintains subwavelength focusing, which means w≤λ. Combining Equations (5) and (6), we obtain the following formula for the relationship of the size parameters.

(7) $$\frac{L_{b}}{h_{1}+h_{2}+f}\geq 1.16$$

Substituting Equation (4) into Formula (7), the focal length and the height of the structure satisfy the following relation.

(8) $$f\leq 2.0\cdot (h_{1}+h_{2})$$

The bottom side length is limited by Equation (9).

(9) $$1.16\cdot \left(h_{1}+h_{2}+f\right)\leq L_{b}\leq 3.5\cdot (h_{1}+h_{2})$$

It can be seen that by introducing the total reflection theorem, we obtain the relationship between the base width and the height. According to the spot size, the limiting condition of the bottom width was obtained.

### 2.2. Modeling Methods

**Figure 2:**
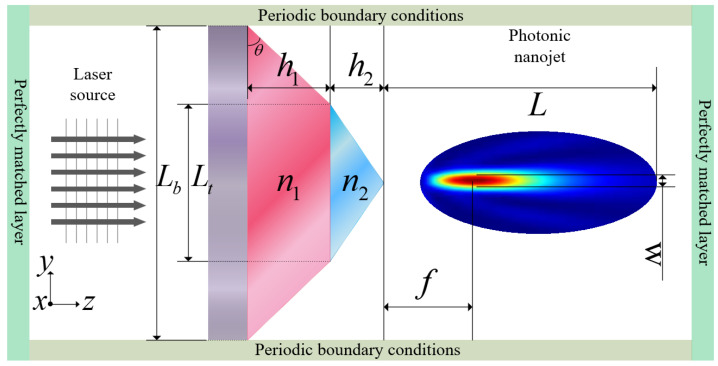
Schematic diagram of the bilayer micropyramid array illuminated by a plane wave.

A three-dimensional (3D) numerical study of the light propagation in a bilayer micropyramid of at the micrometer scale using the FDTD method was implemented. The schematic diagram of the bilayer micropyramid array illuminated by a plane wave is shown in Figure 2.

In the simulation, the bilayer micropyramid array was irradiated by a monochromatic plane wave with wavelength λ=640 nm. The light traveled along the *z*-axis, and the polarization direction traveled along the *y*-axis. The bilayer micropyramid structure contained two different heterogeneous materials. The bottom was a frustum of a microsquare pyramid consisting of silicon nitride (SiN_*x*_), while the top was a microsquare pyramid consisting of silicon oxide (SiO_2_). The refractive indices of the bottom (SiN_*x*_) layer ($n_{1}$) and the top SiO_2_ layer ($n_{2}$) were 2.02 and 1.458 when the wavelength was 640 nm. The substrate was silica glass, while the bilayer micropyramid array was surrounded by air ($n_{air}=1$). The refractive index of the silica glass was 1.4567 (Re-index) and 0 (Im-index) at a wavelength of 640 nm. The thickness of the substrate was set to be more than 6 wavelengths.

In order to achieve a long working distance, the focal length can be set as f≥2λ. Limited by manufacturing, this would cause a stress mismatch between the two layers due to the large thickness. After repeated experiments, the film thickness ($h_{1}$ and $h_{2}$) was set to no more than 2 μm. Considering the height reduction problem caused by the etching ratio, the height of the top structure ($h_{2}$) is usually reduced by about 1/3. In order to be as consistent as possible with the fabrication, $h_{1}$ and $h_{2}$ were 2 μm and 1.3μm. Substituting this into Formula (9), the limited range of $L_{b}$ is as follows, 5.3μm $\leq L_{b}\leq$11.6μm. Taking the height and the ratio of the microstructure into consideration, the base side lengths of the bottom and top structure were obtained, *L*_*b*_ 7.6 μm, $L_{t}=3.8$μm. The structural gap $L_{g}$ was 0.

The perfectly matched layers (PML) were applied on the input and output boundaries of the computational domain. In order to match the situation of the array arrangement, the periodic boundary conditions were set on the other four boundaries. The electromagnetic calculation based on a uniform FDTD mesh of 0.21 nm was proven to be an accurate and efficient method to analyze the interaction of light with the bilayer micropyramid array.

To investigate the effect of TIR on PNJ, we added two types of the parental microstructure not in accordance with TIR theory. The base angle was changed to $18^{\circ }$, because the base angle should be bigger than $29.4^{\circ }$ to maintain the TIR effect. For an obvious comparison with the design that introduces TIR theory, one type kept the same base length with the TIR design, and another type kept the same height as the TIR design. These parameters are shown in the Table 1. The refractive indices of $n_{1}$ and $n_{2}$ were 2.02 and 1.458.

The incident intensity, the maximum intensity of the emergent field, the decay length, the full width at half maximum, and the focal distance of the bilayer micropyramid structure were $I_{0}$, $I_{max}$, *L*, *w*, and *f*, respectively. To quantify the extension of the PNJ formed by the bilayer micropyramid, the decay length *L* was defined as the distance from the apex of the bilayer micropyramid to the point where the intensity drops to two-times value of $I_{0}$ [8,46,47]. *f* refers to the length from the apex of the bilayer micropyramid to the point of $I_{max}$, as mentioned.

### 2.3. Fabrication of the Bilayer Micropyramid Array

**Figure 3:**
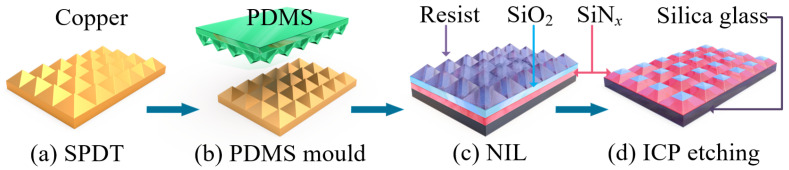
Schematic diagram of the fabrication process. (**a**) copper mould with the micropyramid array was made by SPDT technique, (**b**) polydimethylsiloxane (PDMS) mould was generated to duplicate the micropyramid array, (**c**) NIL mask on the bilayer thin film was formed by nanoimprint technique, (**d**) the bilayer micropyramid array was fabricated by ICP technique.

The bilayer micropyramid array samples were produced via single-point diamond turning (SPDT), nanoimprint lithography (NIL), and inductively coupled plasma (ICP) etching techniques. The schematic diagram of the fabrication process is shown in Figure 3.

Firstly, a micropyramid structure array with a height of 4 μm and a bottom width of 8 μm made of copper alloy with a diameter of 12 mm was used as the mold. Secondly, PDMS was used to replicate the microstructure array. After that, using the nanoimprint lithography resist (MR-I 7030R, Micro resist Technology, Berlin, Germany) as a mask, the micropyramid array was replicated by means of nanoimprint technology. The technical parameters of the three steps refer to the preparation of the homogeneous micropyramid array [45].

Using the film deposition parameters in Table 2, a double-layer film with a top layer of SiO_2_ and a bottom layer of SiN_*x*_ was deposited on the surface of the silica glass. The silicon nitride and silicon oxide films with low stress were obtained by the PECVD method. The thickness and refractive index could be flexibly controlled by adjusting the reaction gas flow ratio, reaction chamber pressure, and Radio Frequency (RF) power. Changing the deposition time, we obtained the required film thickness.

To realize the manufacturing of the bilayer micropyramid array, a step-by-step etching method was creatively proposed. Based on multiple experiments, two sets of etching data were used to realize the preparation of the bilayer micropyramid array. The etching parameters are shown in Table 3.

The thickness of the resist, SiN_*x*_, and SiO_2_ layers was determined by the etch rates ratio of the materials. In order to obtain the designed bilayer microstructure, after experimenting many times, the thickness of the resist, SiN_*x*_, and SiO_2_ layers was 3 μm, 2 μm, and 2 μm, respectively. What is noteworthy is that the thickness of the resist including the pattern was 6 μm in height after the NIL process. To thin the adhesive layer, O_2_ was used for the etching process first. The oxygen etch rate of the adhesive layer was fast, and the resulting aspect ratio change was small. The etching effect of oxygen reduced the thickness of the resist from 6 μm to 3 μm. The aspect ratio of the pyramid structure was reduced from 1:2 to 1:3. At this point, the required height of the mask resist was obtained.

Then, we began the first etching to achieve the transfer of the top structure. Parameter Group 2 was used; only C_4_F_8_ passed through, and the physical bombardment effect was used to complete the structure transfer. After 10.6 min of etching, the nanoimprint resist was completely removed according to the calculation of the etching rate in Table 3.

Next, using Parameter Group 1, SF_6_ and C_4_F_8_ gas were passed in at the same time to etch the SiO_2_ and SiN_*x*_ layers. The etching rate of SiO_2_ was 84 nm/min, and the etching rate of SiN_*x*_ was 221 nm/min because of the physical bombardment effect and the chemical corrosion effect occurring simultaneously. The etching rate of SiO_2_ at the top was much lower than that of SiN_*x*_ at the bottom. Therefore, the ratio of the depth-to-width of the micropyramid could be improved. We continued to etch for 7 min, and the bilayer micropyramid array could be fabricated with the depth-to-width ratio improved using the top SiO_2_ as the mask layer.

The preparation of the bilayer micropyramid array was different from the preparation of the single-layer micropyramid array in the fourth step of ICP etching. Using the two-step etching transfer technique, the bilayer micropyramid array could be successfully fabricated and maintained the desired aspect ratio.

### 2.4. Measurement Setup

**Figure 4:**
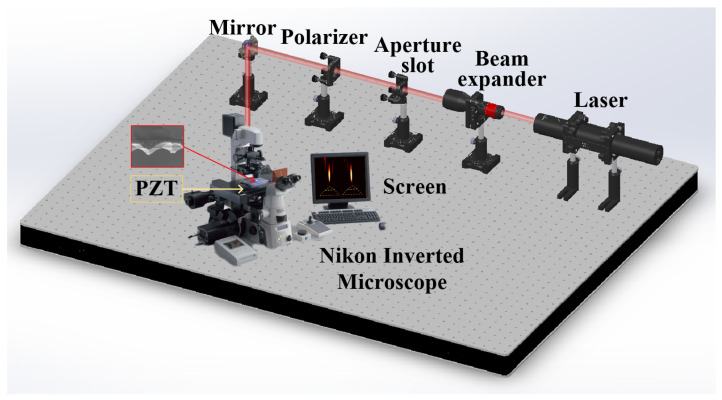
Schematic diagram of the optical setup of the measurement.

The optical field distribution of the bilayer micropyramid array was detected by step-scanning imaging in the *z* direction [48]. The optical setup of the measurement is shown in Figure 4.

The monochromatic light (λ=640 nm) transmitted through the bilayer micropyramid array was collected by the Nikon inverted microscope objective (100×/1.4 Nikon, Tokyo, Japan). The image contours could be further recorded by a charge coupled device (CCD) camera, for which the minimum image size was 0.3μm (Nikon, Tokyo, Japan). The step-scanning imaging was realized with a step of 0.02μm as the highest accuracy in the *z* direction by a piezoelectric transducer (PZT) controller (E-816, Physik Instrument, PI, Karlsruhe, Germany).

The experimental steps were as follows. The BMP-PNJ sample was placed on the surface of the PZT test platform in the form of a downward micropyramid tips. The optical wave transmitted through the bilayer micropyramid array from the above, which would be concentrated in a few wavelengths below the sample. Every time the PZT platform moved a step in the *z* direction, the CCD recorded the light intensity distribution once. At the same time, the sample was linked to the PZT platform. We collected 201 images of light intensity distributions at a step of 0.1μm. After that, the data was extracted and analyzed. The side-view cross-section of the PNJ was obtained by means of tomographic scanning.

## 3. Results and Discussion

### 3.1. Manufacturing Results

**Figure 5:**
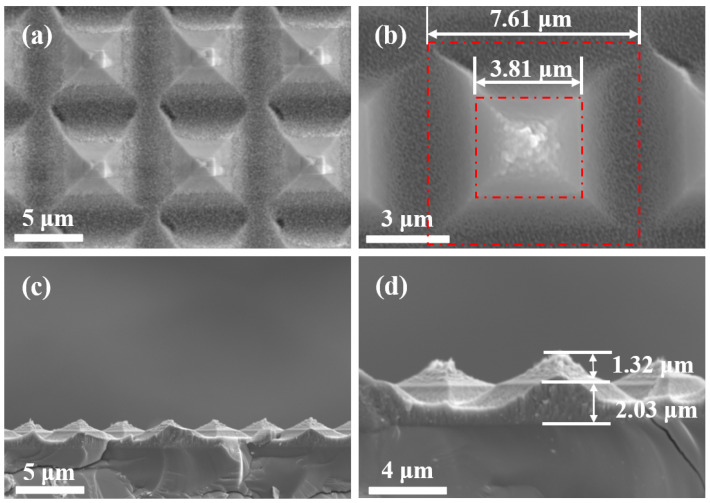
SEM diagrams of the bilayer micropyramid array: (**a**,**b**) is in the top view, and (**c**,**d**) is the cross-section view.

The manufacturing results were consistent with the design, which was due to the step-by-step etching method for the bilayer micropyramid transferring. The results of the manufacturing are shown in Figure 5. The designed and experimental parameters of the fabrication are shown in Table 4.

The base side-lengths of the bottom and top structure were 7.61μm and 3.81μm, and the height was 2.03μm and 1.32μm, which deviated very little from the designed size. The only difference lied in the fabrication compared to the design, where a 0.78μm spacing occurred between the two micropyramids. The red line means the contour line of the layered interface. The two red squares demonstrate the base side-lengths of the bottom and top structure, respectively, in Figure 5. The preparation of the bilayer micropyramid was different from that of the single-layer micropyramid. First, the material of each layer was diverse, which led to a great difference in the anti-etching characteristic of each layer. The bilayer micropyramid array could not be formed in a one-step process with a single etching parameter as in the manufacturing of single-layer microstructures. Second, in order to ensure the continuity of the structure profile between layers, it was necessary to consider the stress matching between the layers. Before the bilayer micropyramid array transfer process, the thickness of the resist, the silicon nitride, and the silicon oxide thin film was 3 μm, 2 μm, and 2 μm, respectively. The thickness matched the ratio of the etch rates, which ensured the preparation of the bilayer micropyramid array in accordance with the design. The manufacturing results proved that the step-by-step etching method was suitable for the preparation of the bilayer micropyramid array.

### 3.2. Characterization of the Focusing Properties

**Figure 6:**
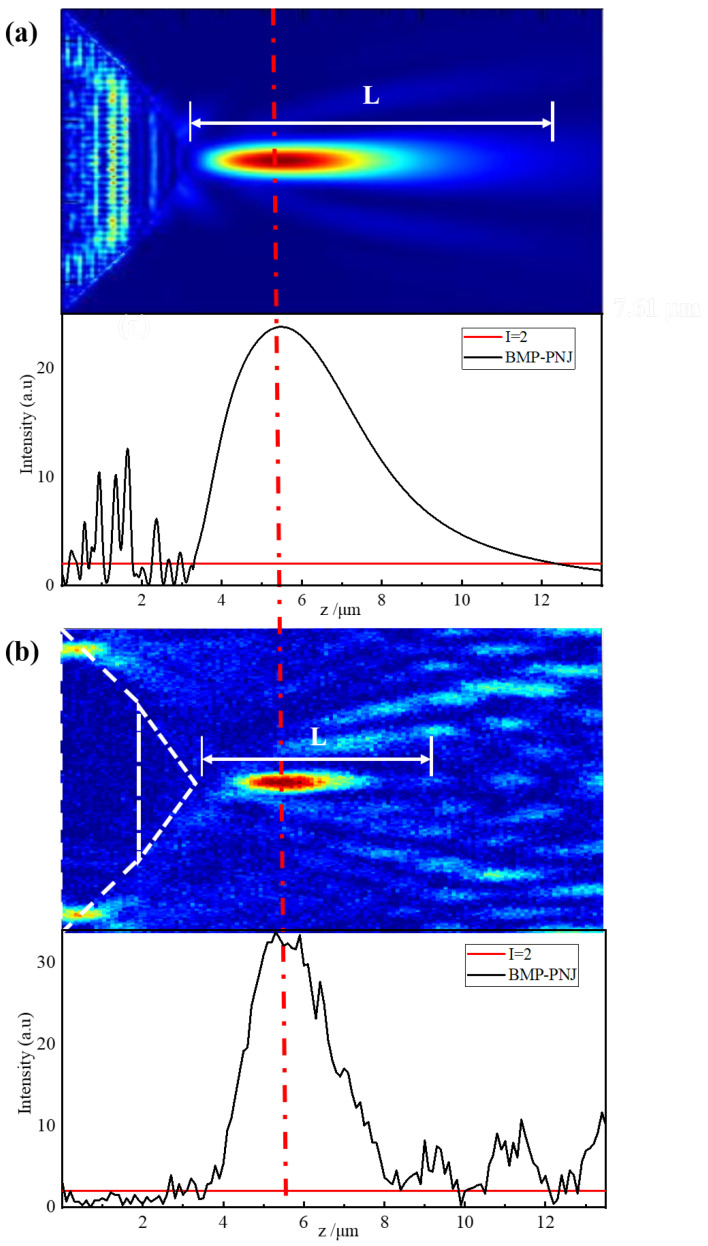
Normalized intensity distribution of the BMP-PNJ along propagation axis (*z*-axis) generated by (**a**) the numerical simulations and (**b**) the actual experiments.

**Figure 7:**
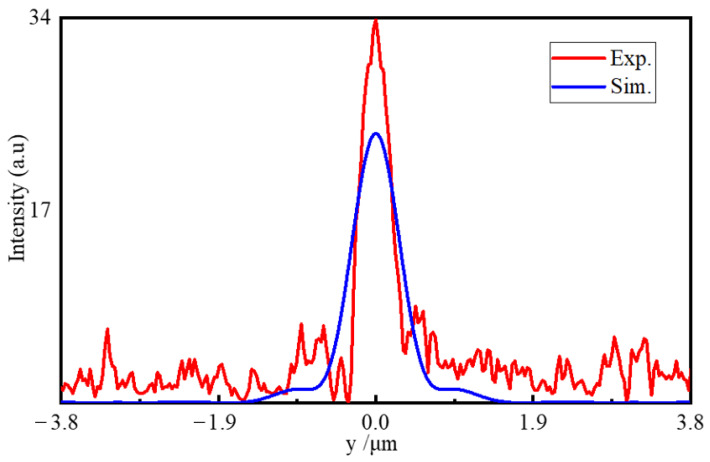
Normalized intensity distribution of the BMP-PNJ along the transversal axis (*y*-axis).

The comparison of the intensity profiles of the PNJ generated from the numerical simulations and the actual experiments is shown in Figure 6 and Figure 7. The maximum light intensity, decay length, FWHM, and focal distance generated from the simulation and experiment are shown in Table 5.

Taking the results in Figure 6 and Figure 7 and Table 5 into account, it simultaneously provided evidence to illustrate that the design followed the total internal reflection theorem, enhancing the intensity of the emergent optical field. Furthermore, the bilayer thin film inside the micropyramid structure extended the field intensity range benefits from the interference effects. All the parameters of the simulation and experiments satisfied the theoretical design values.

The distribution of the normalized intensity along the *z*-axis characterizes the optical modulation of the bilayer micropyramid. The light intensity increased to 33.8-times the initial one and was maintained in a certain area, which was stronger than the intensity of the simulation (23.8-times the initial one), as shown in Figure 6a,b. The decay length of the actual experiments was 10.08 wavelengths, as shown in Figure 6b, which was different from the homogeneous micropyramid whose $I_{max}$ appeared near the apex [23,28,37,49]. The focal distance *f* reached three-times the wavelengths, which means the $I_{max}$ position broke through the locality of the near-field, bringing convenience to the manipulation of microparticles (as shown in Table 5).

The simulated results showed good agreement with the actually measured ones. The experimental position of maximum intensity was basically the same as that of simulation with a deviation of only λ/7. The simulated intensity had a wider range (to 14.22λ), but its intensity was lower than the test value. It should be noted that the measured FWHM value of ~0.6λ was very close to the diffraction limit, which was far better than the simulation result, as shown in Figure 7 and Table 5. The difference in the focusing characteristics came from the replication deviation caused by the manufacturing. The structural gap produced by the actual preparation enhanced the light field coupling effect between the microstructures, which was one reason for the stronger intensity and smaller light spot, as proven by Gao [50]. The simulation calculation pointed out the direction for fabrication and measurement, and the analysis of the simulation results helped explain the formation mechanism of the BMP-PNJ.

**Figure 8:**
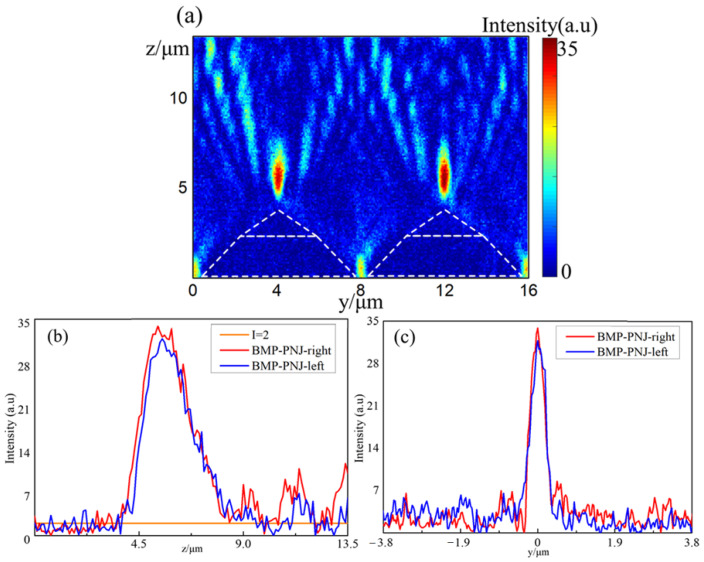
Normalized intensity distribution of the two nearest experimental BMP-PNJs (**a**) along the propagation axis (*z*-axis), (**b**) the decay length, and (**c**) along the transversal axis (*y*-axis).

The experimental images of the two nearest BMP-PNJs are presented in Figure 8. The maximum light intensity, decay length, FWHM, and focal distance are shown in Table 5.

The two nearest bilayer micropyramid structures formed similar focused light field distributions. The difference in the decay length and focal distance were in the range of half a wavelength. Although the values were slightly different, the trend of change was very similar, as shown in Figure 8a–c. It is worth noting that the left BMP-PNJ test showed that it broke the diffraction limit as one distinguishing feature of PNJs. These comparisons showed that although the results were similar, there were still some nonuniformities in the experimental process.

**Figure 9:**
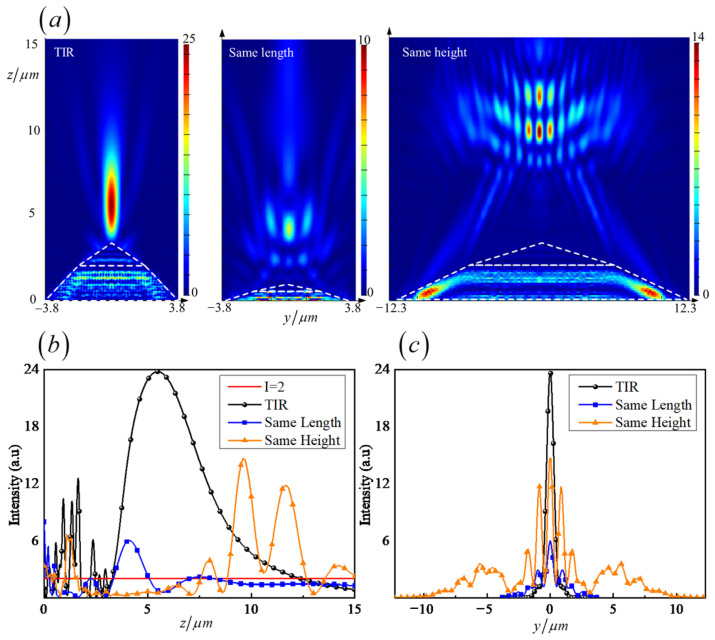
Normalized BMP-PNJ intensity distribution of three types, (**a**) along the propagation axis (*z*-axis), (**b**) the decay length, and (**c**) along the transversal axis (*y*-axis).

The effect of TIR theory can be illustrated by the comparison of the three BMP-PNJ types. The intensity profiles generated from the TIR, for the same length and same height PNJ, are shown in Figure 9. The maximum light intensity, decay length, FWHM, and focal distance are shown in Table 6.

As can be seen from Figure 9a, when the base angle was smaller than the critical angle, which means without the TIR effect, the decay length was reduced to less than one half, and the maximum light intensity was only $\frac{1}{4}$ in the case of the same base width dimensions. When the height of the micropyramid was the same as the TIR design, the decay length and the maximum light intensity both significantly reduced. What is noteworthy is that the focal length was increased nearly threefold. Figure 9b further shows the numerical comparison of the decay length in accordance with TIR theory or not. The sum of the latter two types’ decay lengths was not comparable with that of the TIR-designed PNJ. The FWHMs are demonstrated in Figure 9c. The base length determined the size of the focal spot. When the base length was long enough, there were two noticeable side lobes, which limited the resolution of the BMP-PNJ. Therefore, the use of TIR theory played a significant part in extending and enhancing the photonic nanojet. Table 6 provides the corresponding numerical proof.

**Figure 10:**
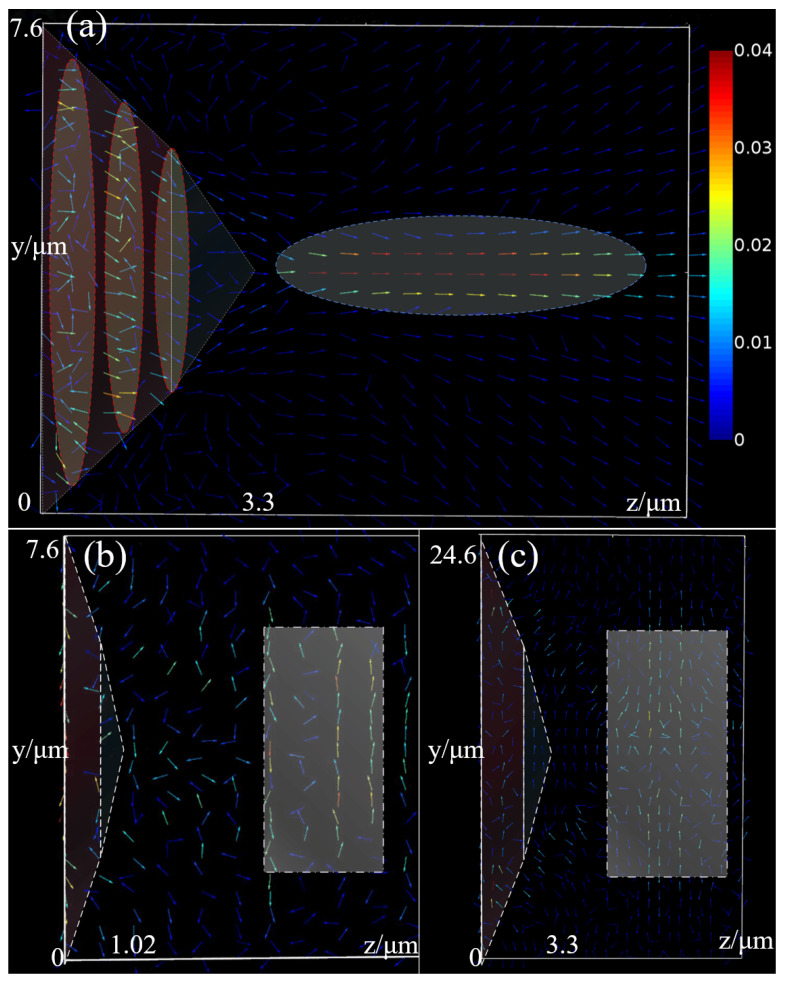
The power flow (time-averaged Poynting vector) plots for the BMP-PNJ. (**a**) TIR-designed, (**b**) same length without TIR, (**c**) same height without TIR.

The power flow (time-averaged Poynting vector) plots for the three BMP-PNJs are shown in Figure 10. When the incident wave entered the microstructure, the propagation direction was changed because of the effect of total internal reflection; even some light waves transmitted laterally, and other light waves converged at the pyramid tip along the sidewall of the structure (as illustrated in the red ellipse). This means that the incident energy was concentrated inside the structure. When the light wave propagated to the interface of the film layer, the incident wave interfered with the reflected wave, thus slowing down the convergence of the power flow and radiating the focusing effect to the far-field (as illustrated in the blue ellipse), which is presented in Figure 10a.

The other two PNJ designs without TIR theory were much weaker in the light intensity and decay length. Many light waves entered the bottom of the micropyramid and then passed out, losing much energy, as seen in Figure 10b,c. There was no obvious interference effect inside the bilayer microstructure due to the lack of the TIR effect converging the waves. Subsequently, the light waves diverged rapidly after transmission, resulting in transverse propagation in the region away from the tip of the micropyramid, which was also quite different from the TIR-designed BMP-PNJ.

Given the above, the use of TIR theory was crucial to obtain the most extended and intense photonic nanojet. Based on the power flow plots, it is obvious that the enhancement of light intensity was caused by total internal reflection. The direction of energy flow also illustrated the effect of interference effects on the extension of the decay length.

## 4. Conclusions

In summary, the BMP-PNJ was successfully designed by the theoretical calculation method, which combined TIR theory and vector diffraction theory. Furthermore, the low-cost and large-area bilayer micropyramid array was also successfully fabricated via the SPDT, NIL, and ICP technologies. The actual optical field distribution coincided with the results of the FDTD simulation. According to the analysis of the intensity and power flow distribution, it is noteworthy that the intensity of the focused optical field was enhanced and the focusing effect radiated to the far-field, which was due to the interference of the optical field at the interface of multiple layers. The intensity of the focused optical field reached 33.8-times that of the initial light; the decay length was achieved as 10.08λ; the FWHM was 0.6λ (close to the diffraction limit). These results indicate that the BMP-PNJ has promising practical application prospects in nanolithography, high-resolution imaging, and optical trapping. The results as a scaled model may be extended to other areas [49,51]. In addition to the refraction, diffraction, and interference effects, the focused light field was also strongly affected by the coupling effect between the microstructures, which will be the focus of further research.

## Figures and Tables

**Table 1:** Design parameters of the BMP-PNJ.

Type	$L_{b}$/μm	$L_{t}$/μm	$h_{1}$/μm	$h_{2}$/μm	θ/°	TIR
TIR	7.60	3.80	2.00	1.30	46.5	Used
Same length	7.60	3.80	0.62	0.40	18.0	Not used
Same height	24.60	12.3	2.00	1.30	18.0	Not used

**Table 2:** Deposition parameters of the silicon nitride and silicon oxide thin film.

	SiH_4_/Sccm	N_2_/Sccm	N_2_O/Sccm	Temperature/°C	RF Power/W	Pressure/Pa	Deposition Rate/nm·min^−1^
1	120	0	60	250	30	220	64.9
2	40	103	0	250	270	67	21.3

**Table 3:** ICP etching parameters.

	SF_6_/Sccm	C_4_F_8_/Sccm	ICP/W	RF/W	Pressure/mTorr	Etching Rate /nm·min^−1^			Ratio of Etch Rates/Resist:SiN_*x*_:SiO_2_
						Resist	SiN_*x*_	SiN_2_	
1	45	7	500	20	15	312.5	221.0	84.0	1.4:1:0.38
2	0	7	1000	100	0.8	283.0	228.0	215.0	1.2:1:0.94

**Table 4:** The designed and experimental parameters of the fabrication.

	$L_{b}$/μm	$L_{t}$/μm	$L_{g}$/μm	$h_{1}$/μm	$h_{2}$/μm	$n_{1}$	$k_{1}$	$n_{2}$	$k_{2}$
Simulation	7.60	3.80	0	2.00	1.30	2.02	0	1.458	0
Experiment	7.61	3.81	0.78	2.03	1.32	2.04	2.2 × 10^−5^	1.462	1.6 × 10^−5^

**Table 5:** Main parameters of the two nearest BMP-PNJs.

Type	$I_{\max}$/(a.u)	*L*/λ	*w*/λ	f/λ
Simulation	23.8	14.2	1.0	3.4
BMP-PNJ-right	33.8	10.1	0.6	3.0
BMP-PNJ-left	31.8	9.6	0.4	3.4

**Table 6:** Main parameters of the BMP-PNJ.

Type	$I_{\max}$/(a.u)	*L*/λ	*w*/λ	*f*/λ
TIR	23.80	14.22	1.00	3.40
Same length without TIR	6.03	6.21	0.95	4.81
Same height without TIR	14.70	5.78	1.91	9.80

## Data Availability

Data is contained within the article. The data presented in this study are available in Section 2 (Materials and Methods) and Section 3 (Results and Discussion).
